# Functional validation of *TERT* and *TERC* variants of uncertain significance in patients with short telomere syndromes

**DOI:** 10.1038/s41408-020-00386-z

**Published:** 2020-11-17

**Authors:** Alejandro Ferrer, Abhishek A. Mangaonkar, Susanna Stroik, Michael T. Zimmermann, Ashley N. Sigafoos, Patrick S. Kamath, Douglas A. Simonetto, Mark E. Wylam, Eva M. Carmona, Konstantinos N. Lazaridis, Steve Peters, Keith Stewart, Eric W. Klee, Eric A. Hendrickson, Mrinal M. Patnaik

**Affiliations:** 1 Center for Individualized Medicine, Mayo Clinic, Rochester, MN USA; 2 Department of Health Sciences Research, Mayo Clinic, Rochester, MN USA; 3 Department of Hematology, Mayo Clinic, Rochester, MN USA; 4 Department of Biochemistry, Molecular Biology and Biophysics, University of Minnesota, Minneapolis, MN USA; 5 Genomic Sciences and Precision Medicine Center, Medical College of Wisconsin, Wauwatosa, USA; 6 Department of Oncology, Mayo Clinic, Rochester, MN USA; 7 Department of Gastroenterology, Mayo Clinic, Rochester, MN USA; 8 Pulmonary and Critical Care Medicine, Mayo Clinic, Rochester, MN USA; 9 Present Address: Lineberger Comprehensive Cancer Center, University of North Carolina at Chapel Hill, Chapel, Hill, NC USA

**Keywords:** Translational research, Haematological diseases, Disease genetics, Genetics research

Dear Editor,

Accelerated shortening of telomeres can induce premature cell senescence that can clinically manifest as bone marrow failure (BMF), idiopathic pulmonary fibrosis (IPF), cryptogenic cirrhosis, nodular regenerative hyperplasia, vascular malformations, immunodeficiency, and structural brain abnormalities, all of which are included under the umbrella term of short telomere syndromes (STSs)^1^. Two main criteria used to diagnose STS include the documentation of shortened telomere lengths (TLs) by a Clinical Laboratory Improvement Amendments (CLIA)-certified flowFISH (fluorescence in situ hybridization) assay^2^ and the presence of pathogenic variants in genes related to telomere maintenance identified through next-generation sequencing (NGS). These genes include, but are not limited to, *hTERT* (human telomerase reverse transcriptase) and *hTERC* (human telomerase RNA component), the two main components of the telomerase holoenzyme complex that is responsible for creating new telomeric DNA (Supplementary Fig. 1)^1^.

In our experience, a pathogenic variant in the coding sequence of a telomere-associated gene can be identified in only 40% of STS cases using current sequencing approaches, with the remaining cases either having no identifiable variant or possessing a variant(s) of uncertain significance (VUS)^1,3^. In addition, in some of these cases, the TL values were not conclusively shortened (i.e., not < first percentile in lymphocytes and/or granulocytes documented by a CLIA-certified FlowFISH assay) complicating the diagnosis. At Mayo Clinic, we established a dedicated BMF clinic in collaboration with the center for Individualized Medicine and the division of Hematology, so as to leverage the latest NGS technologies and functional assays to assist patients with unexplained BMF syndromes, including STS-related marrow failure^3,4^. We have developed a systematic algorithmic approach to assess STS patients that includes TL testing by flowFISH and genomic assessment of STS-related gene mutations using an in-house designed targeted NGS panel (Supplementary Table 1). Through this effort, we have identified 32 patients with an STS phenotype and TLs at or below the tenth percentile. Twenty-two patients (69%) did not have detectable pathogenic variants (in spite of a clinical phenotype) or were found to carry a VUS in STS-related genes (ten patients; 31%). In the latter group, the VUS were located in *hTERT* (four variants), *hTERC* (one patient), *RTEL1* (regulator of telomere elongation helicase 1; two variants), *TINF2* (TERF1-interacting nuclear factor 2; two variants), and *NAF1* (nuclear assembly factor 1 ribonucleoprotein; one variant). Patients with *hTERT* VUS (Table 1 and Supplemental Fig. 2) were selected for three-dimensional (3D) computational modeling and functional interrogation, largely due to the availability of a well-described functional assays, namely the telomerase repeat amplification protocol (TRAP), which can be used to test telomerase activity (Supplemental Methods)^5,6^.

**Table 1:** Table summarizing clinical features, variants, and testing results for patients with a clinical phenotype of a STS.

Case no.	Age/sex	Clinical features	Significant family history (yes/no)^a^	FlowFISH TL (centile length in granulocytes/ lymphocytes)	Gene	cDNA change	Protein change	Protein region	In silico predictions (SIFT/PolyPhen)	CADD score	3D model prediction	Conclusion from TRAP assay	ACMG classification (at the clinical report/after research testing)
1^b^	23/M	Premature graying of hair, macrocytosis, thrombocytopenia, bilateral hip avascular necrosis	Yes	<1st/<1st	TERT	c.2768 C > T	p.Pro923Leu	Reverse-transcriptase domain	Deleterious/probably damaging	24.2	Destabilizes structure	NA	Pathogenic/pathogenic
2	69/F	Premature graying of hair, IPF, anemia, leukopenia	No	10th/10th	TERT	c.3362 C > T	p.Pro1121Leu	C-terminal extension	Deleterious/probably damaging	22.9	Destabilizes structure	Absent telomerase function	VUS/likely pathogenic
3	27/F	Macrocytosis, neutropenia	No	<1st/1st	TERT	c.1765A > C	p.Ile589Leu	None described	Tolerated/benign	5.08	Neutral	Decreased telomerase function	VUS/likely pathogenic
4	19/M	Macrocytosis	No	<1st/<1st	TERT	c.1885G > A	p.Gly629Arg	Reverse-transcriptase domain	Deleterious/probably damaging	23.1	NA	Decreased telomerase function	VUS/pathogenic
5	47/M	IPF, pancytopenia	No	<1st/<1st	TERC	n.238 G > C	NA	NA	NA	NA	Changes TERC organization	NA	VUS/pathogenic

*FISH* fluorescence in situ hybridization, *IPF* idiopathic pulmonary fibrosis, *NA* not applicable, *STS* short telomere syndrome, *TL* telomere length, *TRAP* telomerase repeated amplification protocol.

^a^Significant family history was defined as the presence of one or more first- or second-degree relatives with one or more clinical features characteristic of STSs, such as premature onset of hair graying (age < 30 years), IPF, cryptogenic cirrhosis or nodular regenerative hyperplasia, or unexplained cytopenias.

^b^Clinical history of this case were previously published; however, functional testing results are new.

All patients (Table 1) had BMF with additional STS features (IPF in patients 2 and 5, and premature graying of hair in patients 1 and 2). FlowFISH testing indicated a TL below the first centile in both lymphocytes and granulocytes in all patients, except in patient 2 were the TL was at the tenth centile range (Fig. 1a). NGS testing uncovered *hTERT* variants in patients 1–4 and a *hTERC* variant in patient 5. To increase our interpretive resolution for *hTERT* variants, we created a computational 3D protein model of the human telomerase complex that we used to assess the intra- and inter-molecular interactions within the complex. Our current model includes all amino acids of hTERT and a contiguous section of hTERC with the bound DNA heteroduplex. This model was generated using homology-based methods^7,8^ and the experimentally solved structure of TERT from *Tetrahymena thermophila* [PDB 6d6v^9^]. The human Reverse Transcriptase thumb domain of telomerase was experimentally solved [5ugw^10^] and added to our model using threading. Genomic variants were assessed in their 3D context using FoldX^11^. Recent model fitting to a cryogenic electron microscopy map of human telomerase core particle was published^12^ and was used here in comparison to our current model of full-length TERT.

**Fig. 1:**
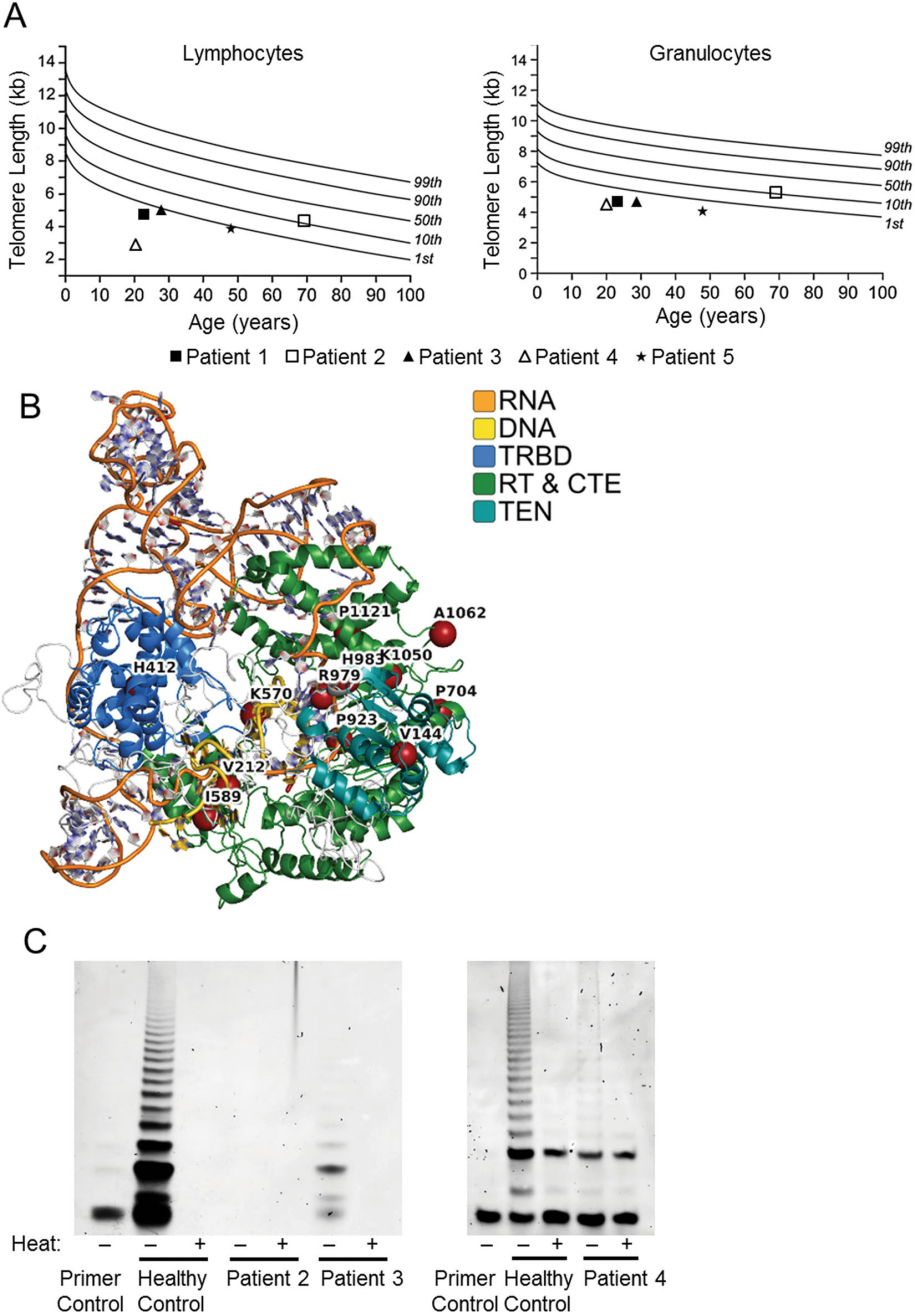
Functional validation of variants of uncertain significance in *hTERT/hTERC*. **a** Mean telomere length in lymphocytes and granulocytes from blood samples measured by flowFISH. All patients presented clinical signs of STS and genetic variants in the holozenzyme telomerase. While most patients (1, 3, 4, and 5) presented clearly shortened telomeres, patient 2 showed fringe values closer to the 10th centile. **b** Recent studies have resolved human telomerase core components, which can be combined with additional data to generate a more specific and comprehensive model for interpreting the effects of telomerase variation. We used molecular modeling to develop a finer resolution of interpretation for telomerase variants identified through clinical genomics sequencing. The nucleotide backbones of RNA and DNA are colored in orange and gold, respectively, and each protein domain is colored distinctly with the intervening loops demarcated in white. Our current model of human telomeres consists of all amino acids of hTERT and a large contiguous section of hTERC. This model was generated based off of homology relationships and energetic refinement. The 3D relationships among amino acids of TERT and nucleotides of TERC enable us to make specific predictions for the impact and effects of genomic variants. **c** Blood protein extracts from patients carrying *TERT* variants were tested using the TRAP assay for telomerase activity. In comparison to healthy individuals used as controls (second lane in each panel), the results indicated absent (patient 2) or diminished (patients 3 and 4) telomerase activity in all samples tested, suggesting pathogenicity of the variants.

When indicated, we used our 3D model to suggest mechanisms of pathogenicity for these variants as follows^1^: *hTERT* c.2768 C > T; p.Pro923Leu (patient 1) is located near the helix required for oligomerization and is adjacent to p.Arg901 and p.Lys902—sites where bona fide loss-of-function mutations have been described (Fig. 1b)^2^. *hTERT* c.3362 C > T; p.Pro1121Leu (patient 2) is present at the end of the reverse-transcriptase thumb domain at a position where the peptide chain crosses back against residues 999–1003. Further, hTERC wraps around one side of this thumb and the DNA winds across the other side, so that any changes to the internal organization/arrangement of this thumb may significantly alter function (Fig. 1b)^3^. The third variant *hTERT* c,1765A > C; p.Ile589Leu (patient 3) affects a residue located in the helix near the hTERC interface possibly affecting stability of this interaction (Fig. 1b)^4^. The fourth variant, *hTERT* c.1885G > A; p.Gly629Arg is located in a loop between the DNA strand of the heteroduplex, and Lys626 and Arg631, both of which make contact with the DNA strand (Fig. 1b).

In addition to this information, further functional testing was pursued for these variants with the exception of *hTERT* c.2768 C > T; p.Pro923Leu (Patient 1), where we considered the evidence already available, including a published functional report, to be strong enough to consider this variant as being pathogenic^13^ (more information in ref. ^3^). For the remaining patients with *hTERT* variants, we evaluated their telomerase activity using the TRAP assay^5^. TRAP semi-quantitatively measures the capacity to elongate a telomere-imitating oligonucleotide via hTERT processing in a patient’s samples over several amplification cycles, akin to a quantitative PCR reaction. The commercially available TRAPeze Telomerase Detection Kit (Supplemental Methods) was employed in peripheral blood mononuclear cells from patients 2–4 and the results indicated decreased telomerase activity in all samples compared to age-matched patient controls (Fig. 1c). This data, together with our 3D-prediction models, allowed us to re-classify these variants as pathogenic or likely pathogenic (Table 1).

The *hTERC* VUS in patient 5 (n.238 G > C) was located in the stem loop in the CD4/5 region and our 3D model analysis indicated a likely reorganization of the stem loop as a consequence of this change, thereby altering the secondary structure of the RNA. Additionally, a previous report had indicated decreased telomerase activity in patients carrying this variant when tested by the TRAP assay^14^. We felt that given the clinical context, this information was sufficient enough to classify this variant as pathogenic without the need for additional testing.

In conclusion, we demonstrate the importance and feasibility of using 3D molecular modeling and functional assays to classify variants identified in the *hTERT* holoenzyme. By using these methods, we were able to provisionally re-classify five VUS identified in patients with STS, an important step forward in mutational nomenclature. Importantly, pathogenic variants in *hTERT/hTERC* comprise 52% of STS-related mutations described in the Human Gene Mutation Database (https://portal.biobase-international.com/hgmd/). For the remaining 48% of mutations that encompass several genes involved in TL regulation, there currently are no reliable functional assays (e.g., assessment of *RTEL1* abnormalities by T-circle detection demonstrates considerable variability). Thus, variants identified in our clinic impacting *RTEL1, TINF2*, and *NAF1* still remain unclassifiable and their causal impact on TL in these patients remains unknown. TRAP assays also demonstrate inherent variabilities between tissue samplings and between individuals within the same age range, limiting the routine implementation of these assays in clinic. The development of cell line-based assays utilizing genetic engineering of variants and assessing their impact on TL could be one potential way of overcoming these limitations. In summary, while we were able to assess the pathogenicity of *hTERT*/*hTERC* variants using 3D modeling and the TRAP assay, a lot more work is needed to help develop accurate in silico approaches and functional testing for variants in other genes regulating TL.

## Supplementary information

Supplemental Figures legends

Supplemental Methods

Supplemental Table 1

Supplemental Figure 1

Supplemental Figure 2
