# Supplementary material for: Functional validation of TERT and TERC variants of uncertain significance in patients with short telomere syndromes
Source: Blood Cancer J. 2020 Nov 17;10(11):120. doi: 10.1038/s41408-020-00386-z (PMC7673118; doi:10.1038/s41408-020-00386-z)
Supplement: Supplementary file 1 — Supplemental Figures legends [file 41408_2020_386_MOESM1_ESM.docx]

**Supplemental Figure 1. Schematic depiction of the main components involved in telomere protection and maintenance.** In the resting state, the 3’ overhang is coiled into an upstream telomere region creating a circular structure (T-loop) stabilized by the different proteins composing the Shelterin complex (multi-colored complex, upper panel). When telomeres need to be extended, the T-loop is dismantled with the assistance of the RTEL1 helicase allowing the telomerase complex (blue-colored complex) to bind to the exposed telomere. The protein component (TERT) then adds additional nucleotides using TERC as a template (bottom panel). Additional proteins (dyskerin, NOP10, NHP2, GAR) are needed for the correct assembly and function of the telomerase complex.

**Supplemental Figure 2. Visual representation of variants described in this report.** The web application ProteinPaint freely offered by St. Jude Hospital (S. Jude reference #SJ-15-0021) was employed to visualize the location of the patients’ variants within the indicated gene. (A) Variants in *hTERT*. (B) Variant in *hTERC*.
