# Supplementary material for: Functional validation of TERT and TERC variants of uncertain significance in patients with short telomere syndromes
Source: Blood Cancer J. 2020 Nov 17;10(11):120. doi: 10.1038/s41408-020-00386-z (PMC7673118; doi:10.1038/s41408-020-00386-z)
Supplement: Supplementary file 2 — Supplemental Methods [file 41408_2020_386_MOESM2_ESM.docx]

**Supplementary methods**

*Telomeric Repeat Amplification Protocol (TRAP)*

Telomerase activity was measured in patient samples using a TRAPeze Telomerase Detection Kit (EMD-Millipore) following the manufacturer’s indications with minor changes. Briefly, protein extract from 5 X 10^6^ PBMCs (peripheral blood mononuclear cells) from each patient was obtained and quantitated using a Bradford analysis following the company’s indications (BioRad). Protein extracts were diluted in CHAPS buffer (EMD-Millipore) to a final concentration of 20 to 300 ng/μL. 10 μL of these samples were heat inactivated at 85°C for 20 min prior to performing the TRAP assay as indicated in the kit instruction manual. Platinum Taq (ThermoFisher Scientific) was used instead of the Taq polymerase provided in the kit, but otherwise the same protocol as described by the manufacturer was followed. The PCR reaction parameters were: 30°C for 30 min; 30 cycles at 94°C (30 s), 59°C (3s) and 72°C (1 min); 72°C for 1 min. After the TRAP assay, samples were resolved in a 10% polyacrylamide gel, and the gels were incubated in a 1:10,000 SYBR GOLD dilution for 30 to 60 min protected from light. Gels were then visualized in a Typhoon Imager.
