# Supplementary figures and images for: Functional validation of TERT and TERC variants of uncertain significance in patients with short telomere syndromes
Source: Blood Cancer J. 2020 Nov 17;10(11):120. doi: 10.1038/s41408-020-00386-z (PMC7673118; doi:10.1038/s41408-020-00386-z)

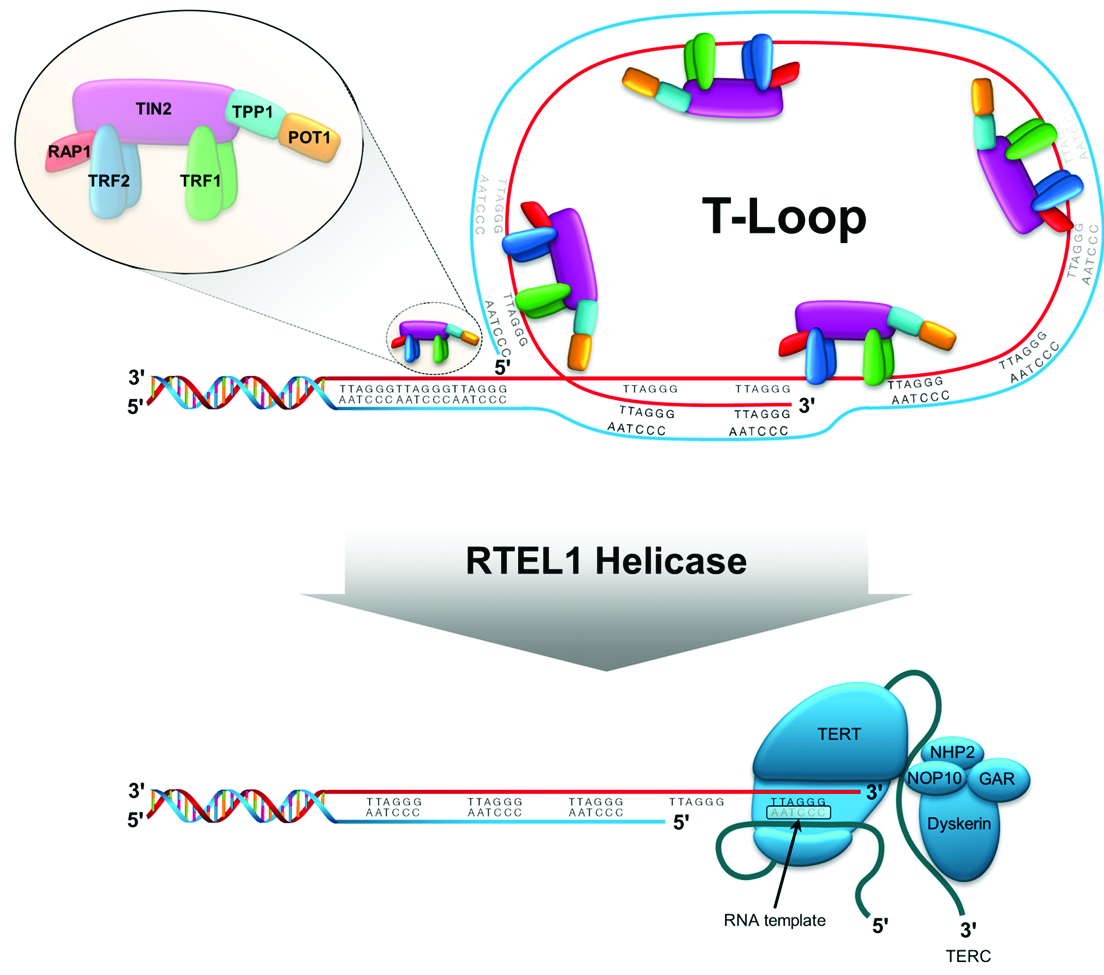

Supplement: Supplementary file 4 — Supplemental Figure 1 [file 41408_2020_386_MOESM4_ESM.tif]
